# A dedicated database system for handling multi-level data in systems biology

**DOI:** 10.1186/1751-0473-9-17

**Published:** 2014-07-10

**Authors:** Natapol Pornputtapong, Kwanjeera Wanichthanarak, Avlant Nilsson, Intawat Nookaew, Jens Nielsen

**Affiliations:** 1 Department of Chemical and Biological Engineering, Chalmers University of Technology, Göteborg, Sweden

## Abstract

**Background:**

Advances in high-throughput technologies have enabled extensive generation of multi-level omics data. These data are crucial for systems biology research, though they are complex, heterogeneous, highly dynamic, incomplete and distributed among public databases. This leads to difficulties in data accessibility and often results in errors when data are merged and integrated from varied resources. Therefore, integration and management of systems biological data remain very challenging.

**Methods:**

To overcome this, we designed and developed a dedicated database system that can serve and solve the vital issues in data management and hereby facilitate data integration, modeling and analysis in systems biology within a sole database. In addition, a yeast data repository was implemented as an integrated database environment which is operated by the database system. Two applications were implemented to demonstrate extensibility and utilization of the system. Both illustrate how the user can access the database via the web query function and implemented scripts. These scripts are specific for two sample cases: 1) Detecting the pheromone pathway in protein interaction networks; and 2) Finding metabolic reactions regulated by Snf1 kinase.

**Results and conclusion:**

In this study we present the design of database system which offers an extensible environment to efficiently capture the majority of biological entities and relations encountered in systems biology. Critical functions and control processes were designed and implemented to ensure consistent, efficient, secure and reliable transactions. The two sample cases on the yeast integrated data clearly demonstrate the value of a sole database environment for systems biology research.

## Background

Systems biology aims to gain insight into complex biological systems by integrating disparate piece of data from various sources and from different levels (such as genome, transcriptome, proteome, metabolome, interactome or reactome), and formulate models that describe how the systems work [1]. The explosive growth in biological and biochemical data is beneficial for systems biology research and it has driven the development of diverse types of biological databases, such as GenBank [2], UniProt [3], SGD [4], HMDB [5], BioGRID [6], KEGG [7], ArrayExpress [8] and GEO [9]. However only 20% of the millions of deposited data in GEO have been referred in other work [9], indicating a bottleneck in utilization of large-scale data. Even though these public repositories ensure easy access to data and hence represent a platform for systems biology research, they were in many cases implemented in isolated groups with a particular purpose in mind. Furthermore, these databases often have distinct data models, different file formats, varied semantic concepts and specific data access techniques [10], and they often contain incomplete data. All in all, those factors make data management and data integration extremely challenging and error-prone.

Attempts have been made to resolve these key issues through the development of numerous data standards (e.g. SBML [11], CellML [12], PSI-MI [13], BioPAX [14], GO [15] and SBO [16]), the implementation of centralized and federated databases (e.g. cPath [17], PathCase [18] and Pathway Commons [19]) and the proposal of design methodologies for software and databases (e.g. I-cubed [20] and [21]). Although, there are still no best practices or solutions to this problem, research and development are underway by making use of current computational technologies, standards and frameworks (see [22] for a review). Here we describe the development of a dedicated database system for handling multi-level data that represents an ongoing endeavor to serve researchers in systems biology and provide alternative solutions for vital issues in data handling, data access and integration of data in a single database. The database system was designed and developed by taking into account: 1) the ability to integrate multi-level data; 2) that biological data are complex, heterogeneous, and dynamic [23]; 3) diversities of resources in terms of data model, semantic heterogeneity, data completeness and data correctness; 4) reusability, extensibility and interoperability of the system; and 5) integrity, consistency and reliability of data in the database. The design of database schema is adapted from BioPAX and implemented based on an object-oriented concept which represents practical information as an object with related attributes and a variety of relationships. This concept is applicable for biological information, which is apparently heterogeneous and sophisticated [24]. The database API was developed in C++ and included a library providing important functions to manage and interact with the system.

To illustrate the integration of multi-level data under a sole database environment, a yeast data repository was developed. The database contains multi-level data of yeast *Saccharomyces cerevisiae* (e.g. genome, annotation data, interactome and metabolic model) from different resources. Data population, data management and data access are managed by the database system. A simple query interface is provided to access the data and related information. Furthermore, two research cases were presented to demonstrate extensibility and efficiency of the database and the underlining database system in facilitating data integration tasks to achieve specific requests.

## Implementation

### Database system design

In order to organize complex data structure efficiently, a specific data model and management library is required to serve the bases of ACID properties including atomicity, consistency, isolation and durability to ensure the correctness of data when used. To control the validity of data changes occurring when the user performs updates to the database, the atomicity concept was applied. In particular, only successful transactions will be committed to the database, otherwise nothing will be committed. Consistency ensures control of data integrity when multiple users are working at the same time. The isolation concept is used for preventing interference between two transactions working on the same data object. The last concept considered was durability, which ensures that committed data will never be lost [25]. The design of the data model follows the basic concepts of a ANSI/X3/SPARC proposed architecture, which uniquely separates the view of the data structure into three layers [26]: 1) an external layer, the first layer of data abstraction in the database system, represents the entities of data to users or applications when querying; 2) a conceptual layer, the second data abstraction layer, represents entities of data that are assembled from the physical layer and are transformed to the external layer as needed; and 3) a physical layer represents the concrete data structure that is implemented in an actual file system and it is only used by the database system. These three layers are set up independently.

**Figure 1:**
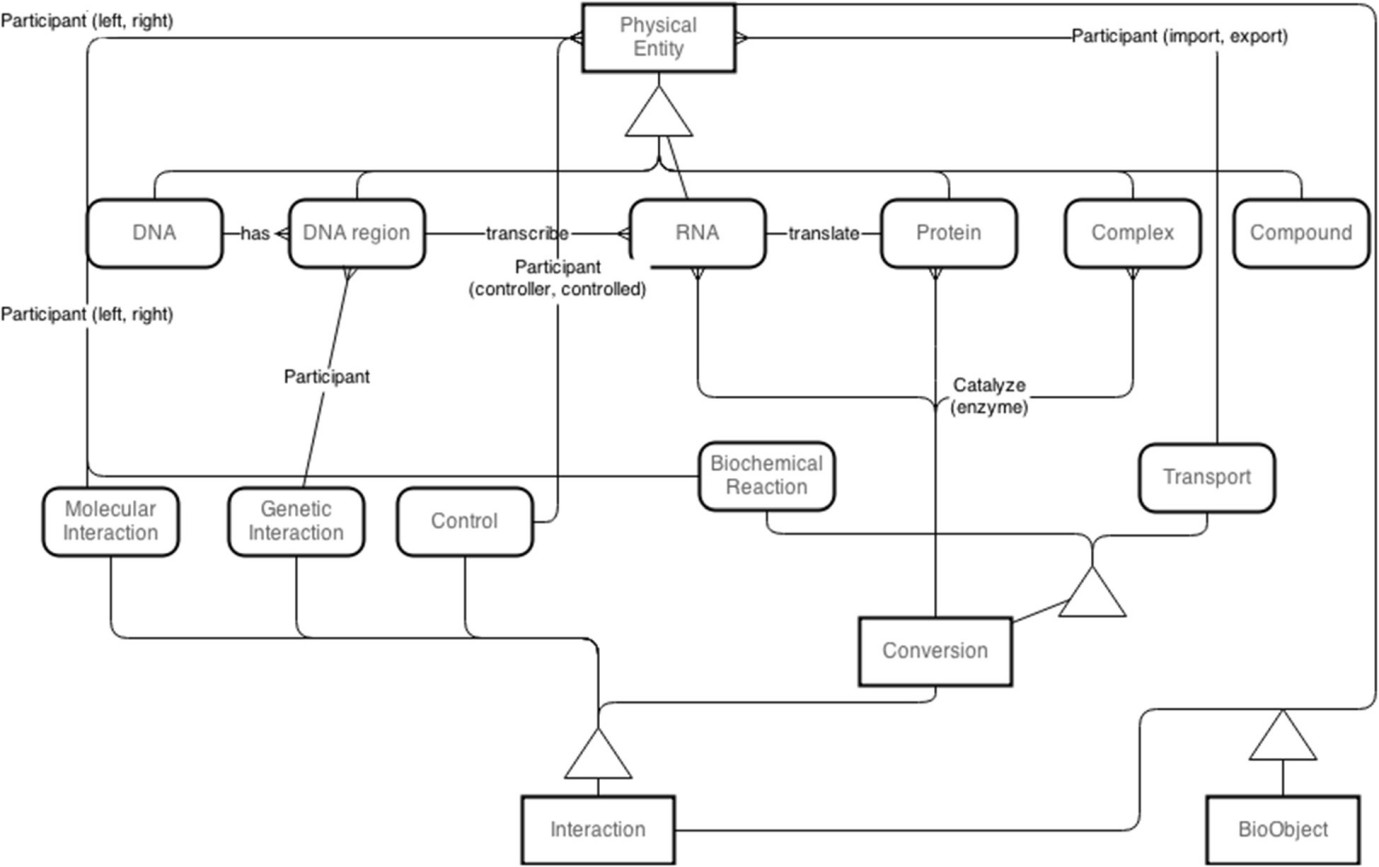
Entity relationship diagram of classes and their relationship.

The conceptual data structure was implemented based on an object-oriented data model. This data model organizes data as a virtual object, which is a group of attributes and their values. The structure of an object is described as a class representing a well-defined state, properties, identity and behaviors of a tangible object that data collected from [27]. A class is comprised of two parts: attributes representing data schema and methods representing abilities to query and transform data of classes. This modeling concept is applicable for assorted and complicated data such as biological data. In this database system, class schema is adapted from Biological Pathway Exchange (BioPAX) and implemented in a database API library to assure the accuracy and completeness of inserted data.The classes were implemented in database API library and were wrapped together, called data wrapper class. This wrapper class was designed by taking in to account the advantage of inheritance feature of object-oriented data model to reduce code implementation and complexity. Therefore, the data classes were inherited hierarchically from the most super class called ‘BioObject’ class as shown in Figure 1. The ‘BioObject’ class was implemented with common attributes such as object id, names, function, cross-references as well as data query and validation methods that share among data wrapper classes. Wrapper classes were separated into two major classes: PhysicalEntity for storing in formation of tangible biological entities and Interaction for storing interaction among PhysicalEntity objects, as shown in the second level sub-classes in Figure 1.

The PhysicalEntity sub-classes, derived from BioObject, support molecular entities including small molecules (SmallMolecule class), DNA molecules (DNA class), genes (DNARegion class), RNA molecules (RNA class), proteins (Protein class) and molecular complex (Complex class) data. The Interaction subclasses, another BioObject derived class, support biological reactions and transport (Conversion class), molecular interactions (MolecularInteraction class), genetic interactions (GeneticInteraction class) and control interactions (Control class). Details of each data wrapper class are elaborated in Additional file 1. Relationships among the sub-classes follow real relations of biological objects to support the data integration of multilevel data as shown in Figure 1. With this data model, reliability of data with its relationship is maintained by data classes themselves, but integrity and consistency are maintained by create, read, update and delete (CRUD) function of the library as described in Additional files 2 and 3.

The instances of the data classes are managed as documents classified by property “type” and pooled together in a document collection, whereas relationships between objects are separated from their own instances and pooled in another document collection to improve the efficiency of managing high complexity relationship of data. In order to optimize query time, an indexing system was applied in common query fields.

### Global system architecture

**Figure 2:**
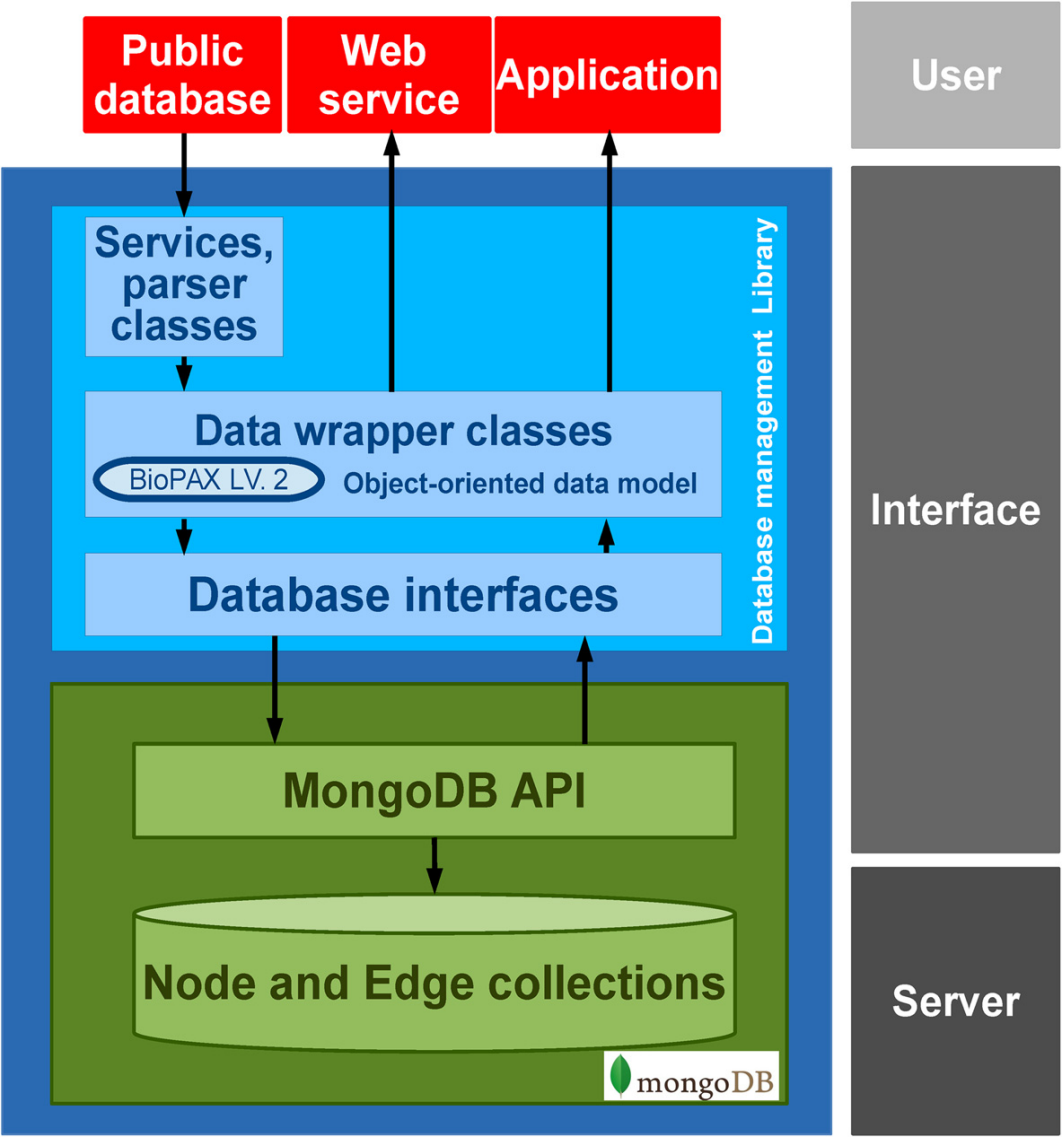
Database management system architecture.

The database system was developed based on the MongoDB library (http://www.mongodb.org), thus the underlying data structure is a document-oriented data model. MongoDB was chosen on account of: 1) the database system can easily be scaled out allowing a modern data management approach such as data centric architecture can potentially be applied to this database system [28,29]; 2) it is possible to change the data schema of the conceptual data layer implemented in the API library [28]; and 3) the MongoDB supports large file storage [29] for storing data such as gene expression data or sequencing reads. However, the schema free property of MongoDB allows storing unstructured data into the database, this might cause data inconsistency. Therefore data wrapper classes in object-oriented data model were implemented in the database API library as interface between developers to the MongoDB to ensure consistency of the stored data.The overview of the system architecture is shown in Figure 2. As the base of the system, the physical layer is managed by a document-based management system, the MongoDB, which contains the necessary interfaces; such as an interactive shell and web services. However, the MongoDB is not designed to manage a specific data structure, especially with complex relationships, and does not have features to control relationships among data objects and this may cause problems in data integrity, consistency and reliability. The database system library was therefore implemented as a core of the system, providing vital functions to manage transactions between developers and the system, and this makes it easy to populate and transform data.

## Results and discussion

### Applications on a yeast data repository

Given that yeast *S. cerevisiae* is a widely used model organism with abundance of genome-scale information and datasets e.g. protein-protein interactions (PPI), transcriptional regulation interactions (TRI), protein kinase interactions (KI), genome-scale metabolic model and gene annotations, integration of data from these different data sources and levels can help to gain new understanding of complex cellular systems.

We therefore developed a yeast data repository as an integrated database that contains various data of yeast. Two types of applications were built on top of the repository. One is a simple web search to query about specific biological objects. The other is additional javascripts for conducting two different research cases which utilize various data in the database to achieve the goals. Those applications are available online at http://atlas.sysbio.chalmers.se:8082. Our intention is to demonstrate efficiency of integrating data under a solitary database environment to help systems biology research rather than to present novel discoveries. As we focus on how the database system is applied, not all features of the database system are illustrated. Specific scripts were implemented to query the data stored in the database.

### Data population and implementation

The yeast data repository comprises of different kinds of biological data such as genome, reactome, interactome and annotations. These data were downloaded in tab delimited or XML format from different repositories (see Table 1). The data were parsed and populated into the database using the parser library in the database system. Each biological molecule (e.g. DNA strand, gene, transcript and protein) corresponds to a specific object in the database. A unique id was assigned to each object and properties associated with it were also stored such as name, primary data source and external references.

**Table 1:** Data in Yeast data repository and sources

**Biological entity**	**Physical entity**	**Data source**	**Amount**
Chromosome	DNA	NCBI GenBank [2]	17
Gene	DNA region	Ensembl [30]	7126
RNA transcript	RNA	Ensembl [30]	7126
Protein	Protein	UniProt [3]	6617
Compound	Small molecule	iTO977 [31]	484
Biochemical reaction	Biochemical reaction	iTO977 [31]	717
Protein-protein interaction	Molecular interaction	BioGRID [6]	72453
Transcriptional regulation interaction	Control	YEASTRACT [32] and [33]	48548
Kinase interaction	Control	[34]	1333
Phosphorylase interaction	Control	[34]	254

In general, biological molecules are related to the molecule in different type (e.g. reaction performed by proteins, proteins translated from transcript, transcripts transcribed from genes and genes are on chromosome). Similar to a biological network, relationships in the database were designed in accordance with real biological phenomena. To insert an object into the database, it is required that such a relation is known. The relational reference is added together with the object and the database system will create a relation object corresponding to that relation pair. These relation objects were used in the cases below to search and explore relationship between one biological object to another. The biological and relation objects were populated separately into two collections: biological collection and relation collection. There were 144,675 documents of biological objects and 268,630 documents of relation objects.

The database system provides a practical library where each object type in the final database corresponds to a C++ object. This allows the user to fully populate the object before inserting it into the database. The database system ensures that all required data is set and pre-forms the task of inserting the object in the database. The task for the user simply becomes the task of gathering the required data, populating the object with the data and inserting the object. For each required data there exists a function such as addname and setlength to add the data to the object.

### Web interface

An online web interface was developed containing links to each application: a simple query interface and a page for case demonstration. The current version allows searching for different object types such as genes, proteins, small molecules, biochemical reactions and interactions with search results that include essential objects related to the queried object. On Cases page, it comprises interactive commands used to compile the two research cases described below.

### Case 1: Detecting the pheromone pathway in protein interaction networks

Signaling pathways transmit signals from one part of the cell to another part through a cascade of protein interactions and protein modifications. Cells organize cellular changes such as transcriptional programs in response to different stimuli. The yeast mitogen-activated protein kinase (MAPK) pathways are signaling pathways that have been extensively studied including pheromone response, filamentous growth, high osmolarity response and maintenance of cell wall integrity [35]. These pathways are activated by sensing stressors of protein sensors or binding of receptors to the stimuli, which in turn triggers MAPKs via a series of phosphorylations. Active MAPKs phosphorylate different targets such as protein kinases, phosphatases and transcription factors (TFs), consequently controlling cell cycle, cellular metabolism and gene expression [36]. The pheromone response pathway is activated by binding of pheromones α- and a-factor to the protein receptors Ste2 and Ste3, respectively. The signals from these membrane receptors are transmitted via sequential binding and phosphorylation reactions of MAPK cascades to TF Ste12 that subsequently activate downstream genes.

In this case, we mined the pheromone pathway segment from PPI networks where both Ste2 and Ste3 were the starting proteins and Ste12 was the ending point. However, this could result in an excessive number of candidate pathways. Several computational methods have been implemented for integrating PPIs and gene expression data or GO annotations to constraint the search [37]. To simplify the case, we integrated only PPI and GO annotation data for finding the pathways. Specific gene ontology terms (GO) in Table 2 were recursively used as constraints to eliminate proteins that are not relevant to the pheromone response pathway and consequently exclude the interactions among those proteins.

**Table 2:** GO terms used for filtering proteins

**GO ID**	**GO term**
GO:0019236	Response to pheromone
GO:0000750	Pheromone-dependent signal transduction involved in conjugation with cellular fusion
GO:0000185	Activation of MAPKKK activity
GO:0071508	Activation of MAPK activity involved in conjugation with cellular fusion

**Figure 3:**
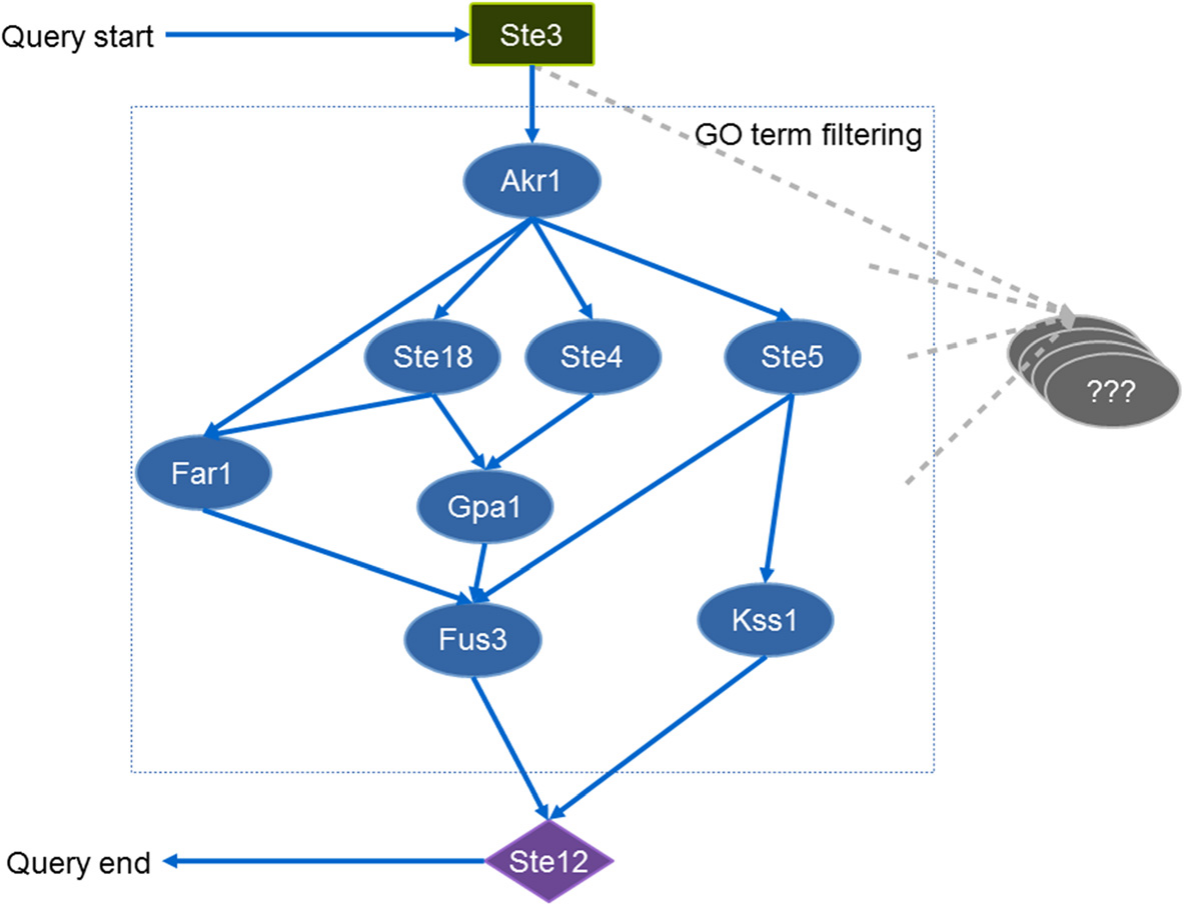
**Diagram of the pheromone pathway and query steps.** Ovals are queried proteins in which blue ovals are included in the pathway while grey ovals are filtered out by GO terms. A green rectangular is the starting node whereas a purple diamond is the ending node where the search stops.

The resulting pathway contains Ste3 as a starting protein and TF Ste12 as an ending node (Figure 3). Key proteins in the pheromone pathway (e.g. Ste4, Ste5 and Fus3) were partially found in comparison to the pheromone pathway from literature [36]. This is because of: 1) the completeness of PPI data; 2) the method used for filtering unrelated proteins; and 3) the number of path lengths to search. As it is beyond our scope, we simplified the pathway construction by using only GO terms as the filters and searching with short path length (3 path lengths). The paths from Ste2 could not be identified. This is because PPI data of Ste2 could not be populated to the database. BioGRID PPI data use gene identifiers (e.g. YNR074C) for protein participants. These gene identifiers have to be mapped to UniProt protein identifiers before populating to the database. However, Ste2 gene was mapped to two proteins which conflict with data propagation rules where PPIs are represented in binary relationship. Thus whole Ste2 interactions were automatically excluded. This case highlights strict restrictions of the data population API in the database library which do not allow data population of conflict information to ensure data integrity.

### Case 2: Finding metabolic reactions regulated by Snf1 kinase

Upon sensing availability of nutrients, cells undergo transcriptional, metabolic and developmental changes in order to survive under a particular nutritional state. In yeast, through complex signaling and regulatory networks, it can grow on a wide variety of nutrients e.g. glucose, galactose, glycerol and nitrogen sources. Key components in these networks include Ras/protein kinase, Snf1 and target of rapamycin complex I (TORC1) [38]. The protein kinase Snf1 is a member of the AMP-activated protein kinase (AMPK) family, which serves as a global energy regulator to ensure metabolic homeostasis of the cells. Under glucose limited condition, it allows the cells to use alternative carbon sources by regulating a set of TFs and genes in several metabolic processes including gluconeogenesis, glyoxylate cycle and β-oxidation of fatty acids [39]. In addition, Snf1 also participates in other processes such as ion homeostasis, general stress response, carnitine metabolism, pseudohyphal growth and ageing [39]. As Snf1 plays an important role in controlling many metabolic processes, we present how processes both directly and indirectly regulated by Snf1 can be retrieved from the database by integrating data from different levels.

**Figure 4:**
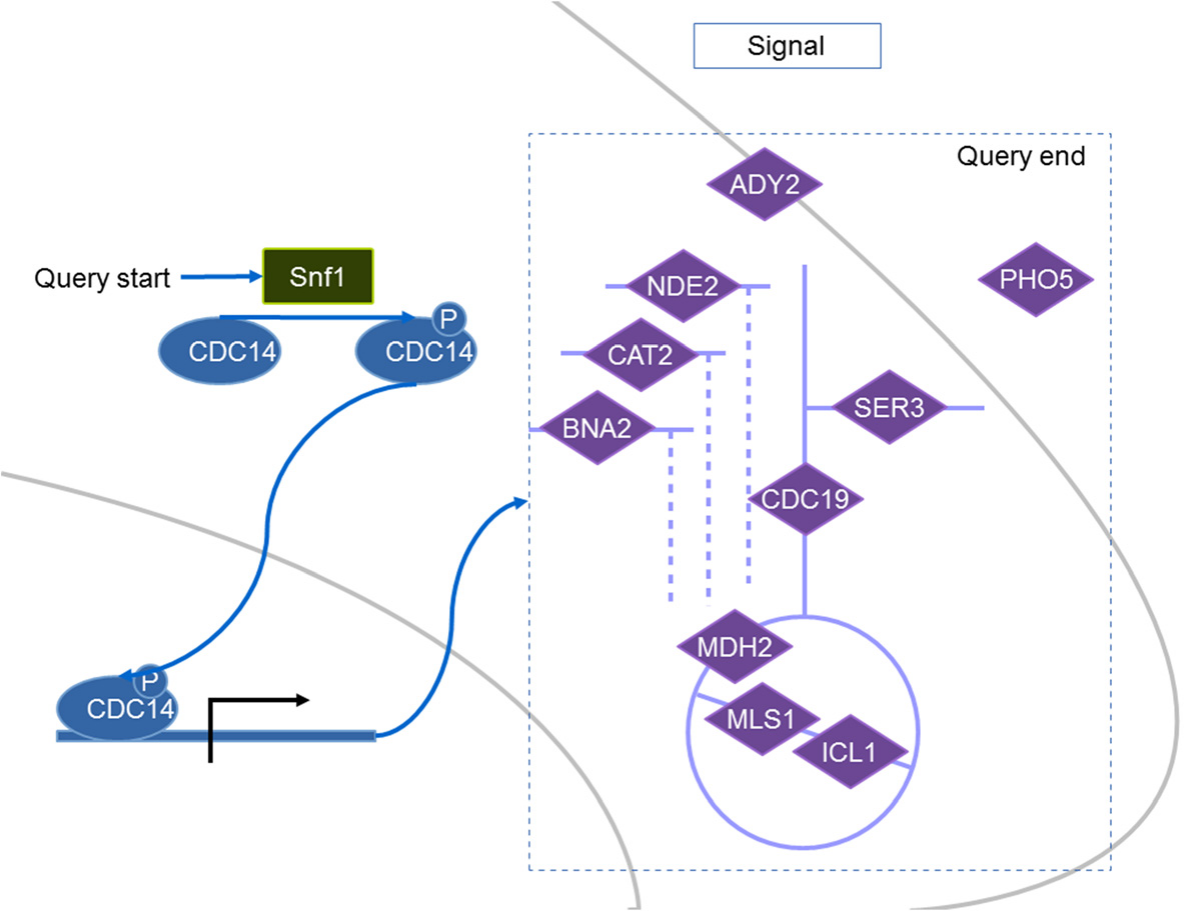
**Diagram of the metabolic genes regulated by Snf1 through TF Cdc14.** A green rectangular is the main gene to start the query, a blue oval is the TF and purple diamonds are metabolic genes.

The *SNF1* gene encodes the Snf1 catalytic subunit which regulates expression of several genes through a variety of TFs. To identify a list of metabolic reactions that are regulated by Snf1, the Snf1 protein is therefore used as a main molecule to construct the query. The first achievement is the identification of TFs, phosphorylated by the Snf1. This was done by querying for the protein targets of Snf1 from the KIs. Then with the TRIs we can retrieve the target genes of those substrates of Snf1 which are acting as TFs. From this list of the target genes we further retrieved biochemical reactions where they are involved in.The queried result is illustrated in Figure 4. From 1333 KIs, we found Cdc14 as the substrate of Snf1 that transcriptionally regulates several metabolic genes involved in the glyoxylate cycle, amino acid biosynthesis, glycolysis/gluconeogenesis, acetate transport and oxidative phosphorylation.

## Conclusions

Here we present a dedicated database model design for handling data in systems biology. It allows and supports crucial tasks in this area including integration and analysis of multi-level data, modeling of cellular pathways and collecting biological network data. In the database design, we have used a basic three layer approach to allow independent and effective implementation or changes at each data layer. The C++ library provides essential classes and services for communication among the layers. The basic properties of the database system, ACID, are responsible for providing specific functions and control processes in the library such as ”insert”, ”remove”, ”update” and ”query” to ensure that database transactions and the data inside are consistent, reliable and not corrupted. An object-oriented concept was adopted for the design and implementation of the database schema because it represents real world information as an object with related attributes and a variety of relationships. It can make the manipulation of this object and its related data easy, straightforward and relatively fast. In addition, the concept is applicable for capturing and reflecting biological information that is apparently heterogeneous and sophisticated [24]. The major design of the conceptual data structure that characterizes data in systems biology was adapted from the BioPAX ontology. Among standards, such as BioPAX, SBML and PSI-MI, for representation of biological pathway data, the main structure of them is fairly similar but BioPAX is the most general [40]. It describes biological objects in a class hierarchy, has explicit use of relations among entities and covers most of the molecular entities in biological pathways. By realizing usages of different standard formats, we included the parser classes in the library. These classes support standard formats that are generally used in most biological databases to accommodate integration of data from different sources to the database and to enhance extensibility of the system.

The database system was applied for establishing the yeast data repository, which represents an integrated platform for performing efficient systems biology research. Two applications were developed showing that building additional applications on a single database environment administrated by the dedicated database system is feasible and convenient. It should be noted that correctness and completeness of results from both research cases are not the main concern in this study, since they are depended on the quality and the availability of data sources. However the restricted control processes and functions in the database API library were designed to ensure integrity and reliability of data in the database.

We believe that the proposed database system shows an extensive attempt to serve and solve complex data handling and integration in systems biology by following and using different standards and technologies. It gives users the ability to extend and personalize the views of data through additional applications and ensures the integrity, consistency and reliability of data in the database.

## Availability and requirements

• Project name: A dedicated database system for handling multi-level data in systems biology.

• Project home page: http://atlas.sysbio.chalmers.se:8082.

• Operating system(s): Platform independent.

• Programming language: C++, php.

• Other requirements: Web Browser.

• Any restrictions to use by non-academics: none.

## Competing interests

The authors declare that they have no competing interests.

## Authors’ contributions

NP and KW designed the database system. NP designed and coded the main library. KW designed and coded the parser class. AN populated the data and implemented the web interface. JN and IN conceived the project. NP, KW and AN wrote the paper and all authors edited it. All authors read and approved the final manuscript.

## Supplementary Material

Additional file 1 Description of data wrapper classes. Click here for file

Additional file 2 **CRUD functions.** Details of Create, read, update and delete (CRUD) function implemented in the system library. Click here for file

Additional file 3 Flow of activities in each function: A) Create; B) Delete; C) Update; and D) Read. Click here for file
