# Supplementary material for: A dedicated database system for handling multi-level data in systems biology
Source: Source Code Biol Med. 2014 Jul 10;9:17. doi: 10.1186/1751-0473-9-17 (PMC4106218; doi:10.1186/1751-0473-9-17)
Supplement: Additional file 1 — Description of data wrapper classes. [file 1751-0473-9-17-S1.pdf]

Description of data wrapper classes

Class name: **BioObject**
Inherit from: -
Attribute list:

- names – storing object names
- type – storing object type
- functionAnnotation – storing functions of objects
- dataXref – storing cross-references

Method list:

- addSynonym – to add a synonym to names attribute
- addAnnotation – to add a functional ontology to functionAnnotation attribute
- addDataXref – to add a cross-reference to dataXref attribute

Class name: **PhysicalEntitiy**
Inherit from: BioObject
Attribute list: inherit from super class and

- cellularLocation – storing sub-cellular localization information

Method list: inherit from super class and

- addCellularLocalization – to add a sub cellular localization information to cellularLocation attribute

Class name: **SmallMolecule – Storing chemical compound information**
Inherit from: PhysicalEntity
Attribute list: inherit from super class and

- inchi – storing an InChI structure of a compound
- inchiKey – storing an InChIKey
- formula – storing a chemical formula
- smiles – storing a SMILES structure description

Method list: inherit from super class and

- setInChi – to set a InChI string to inchi attribute
- setInChiKey – to set a InChIKey string to inchiKey attribute
- setFormula – to set a formula string to formula attribute
- setSMILES – to set a SMILES structure description to smiles attribute

Class name: **DNA – storing DNA molecule information (Chromosome)**
Inherit from: PhysicalEntity
Attribute list: inherit from super class and

- length – storing length of DNA molecule
- source – storing biological source information of DNA molecule

Method list: inherit from super class and

- setLength – to set length attribute
- setSource – to set source attribute

Class name: **DNARegion – storing information of a region on DNA molecule (Gene etc.)**
Inherit from: PhysicalEntity

Attribute list: inherit from super class and

- position – storing start and stop positions of the region

Method list: inherit from super class and

- setPosition – to set position attribute
- setDNASource – to set relation to DNA object

Class name: **RNA – storing RNA molecule information (mRNA etc.)**
Inherit from: PhysicalEntity
Attribute list: inherit from super class and

- position – storing start and stop positions of a transcribe based on position on chromosome

Method list: inherit from super class and

- addPosition – to add start and stop positions to position attribute
- setDNARegionSource – to set relation to DNARegion object

Class name: **Protein – storing protein molecule information (enzyme etc.)**
Inherit from: PhysicalEntity
Attribute list: inherit from super class and

- kineticParameter – storing kinetic parameters

Method list: inherit from super class and

- addRNASource – to add relations to RNA objects
- addSpecificKineticParameter – to add kinetic parameter

Class name: **Complex – storing protein complex information**
Inherit from: PhysicalEntity
Attribute list: inherit from super class and

- kineticParameter – storing kinetic parameters

Method list: inherit from super class and

- addComplexMember – to add relation to protein object that make a complex
- addSpecificKineticParameter – to add kinetic parameter

Class name: **Interaction – storing interaction information**
Inherit from: BioObject
Attribute list: inherit from super class and

- interactionKey – storing a specific hash key for an interaction object

Method list: inherit from super class and

- setInteractionKey– to set interactionKey attribute

Class name: **Control – storing control interaction information**
Inherit from: Interaction
Attribute list: inherit from super class and

- controlType – storing control type such as activate, inhibit
- phenotype – storing phenotypes of this control object

Method list: inherit from super class and

- setController – to set controller object from PhysicalEntity
- setControlled – to set controlled object from PhysicalEntity
- addControlType – to add control type to controlType attribute

- addPhenotype – to add phenotype of this control interaction

Class name: **GeneticInteraction – storing genetic interaction information**
Inherit from: Interaction
Attribute list: inherit from super class and
- interactionType – storing interaction type
- phenotype – storing phenotypes of this control object

Method list: inherit from super class and
- addInteractionType – to add control type to interactionType attribute
- addPhenotype – to add phenotypes of this interaction
- addMember – to add relations to member objects of interaction

Class name: **Conversion**
Inherit from: Interaction
Attribute list: inherit from super class and
- kineticLaw – storing control type such as activate, inhibit
- spontaneous – storing phenotype of this control object

Method list: inherit from super class and
- addEnzyme – to add a relation to enzyme that catalyze this conversion
- addKineticLaw – to add kinetic law
- setSpontaneous – to set spontaneous attribute to be true or false

Class name: **BiochemicalReaction – storing biochemical reaction information**
Inherit from: Conversion
Attribute list: inherit from super class and
- conversionDirection – storing directionality of reaction

Method list: inherit from super class and
- addLeft – to add relations to biological molecules which are substrates of reaction
- addRight – to add relations to biological molecules which are products of reaction

Class name: **Transport – storing transport reaction information**
Inherit from: Conversion
Attribute list: inherit from super class
Method list: inherit from super class and
- addImport – to add relations to biological molecules which are imported by reaction
- addExport – to add relations to biological molecules which are exported by reaction
