# Supplementary material for: A dedicated database system for handling multi-level data in systems biology
Source: Source Code Biol Med. 2014 Jul 10;9:17. doi: 10.1186/1751-0473-9-17 (PMC4106218; doi:10.1186/1751-0473-9-17)
Supplement: Additional file 2 — CRUD functions. Details of Create, read, update and delete (CRUD) function implemented in the system library. [file 1751-0473-9-17-S2.pdf]

Additional file 1

Create, read, update and delete (CRUD) function

In general, most of noSQL DBMSs are lagging the ACID properties. As well as in MongoDB it only provides the A-atomicity operation to single document and D-durability using a journaling system. To maintain integrity, consistency and reliability of data in the database during the committing process and to ensure that most transactions follow the ACID principle, all activities in the database have to be done using the specific functions from the system library. Four basic functions were provided to manage general activities inside the database system as shown in Additional file 2.

Create (in function database::insert)

To avoid duplication, data insertion begins with comparison between cross references from “dataPrimarySource” and “xref” field in an object and the index of the “object” collection. If there is a match, the system will give an error message to the user and terminate the insertion process. Afterwards, relation documents will be generated. If there is no error, an object document will be generated and inserted to the database following by the atomic insertion of relation documents. As previously mentioned, the database system does not support multiple document transaction across the collections. During this “insert” operation, the system needs to insert both the “object” document and related “relation” documents. To maintain ACID properties, if the atomic insertion of relation documents results in an error, the object document added previously will be deleted. The insertion process will be terminated following this.

Delete (in function database::delete)

At the beginning, all objects are obtained from a query string provided by the users. Each “object” document will be masked as a temporary document and, subsequently, related relationship documents will be removed from the “relation” collection with an atomic transaction. At the end, the temporary document will be removed from the database. To maintain ACID properties, if there is an error during

the process of removing relationship documents, the object document will be returned back to the database.

Update (in function database::update)

Update transactions start with masking an original document to be a temporary one followed by inserting the updated object document into the database. If the insertion process fails, the original version of the document will be returned to the database. Otherwise, it will be removed.

Read (in function database::query)

This function uses the query engine of the database system to obtain objects from a submitted query string. Related relationship documents will be queried, and combined with an object document. This query function also provides a process to format the resulting documents to be elements of the data wrapper class.
