# Supplementary figures and images for: A dedicated database system for handling multi-level data in systems biology
Source: Source Code Biol Med. 2014 Jul 10;9:17. doi: 10.1186/1751-0473-9-17 (PMC4106218; doi:10.1186/1751-0473-9-17)

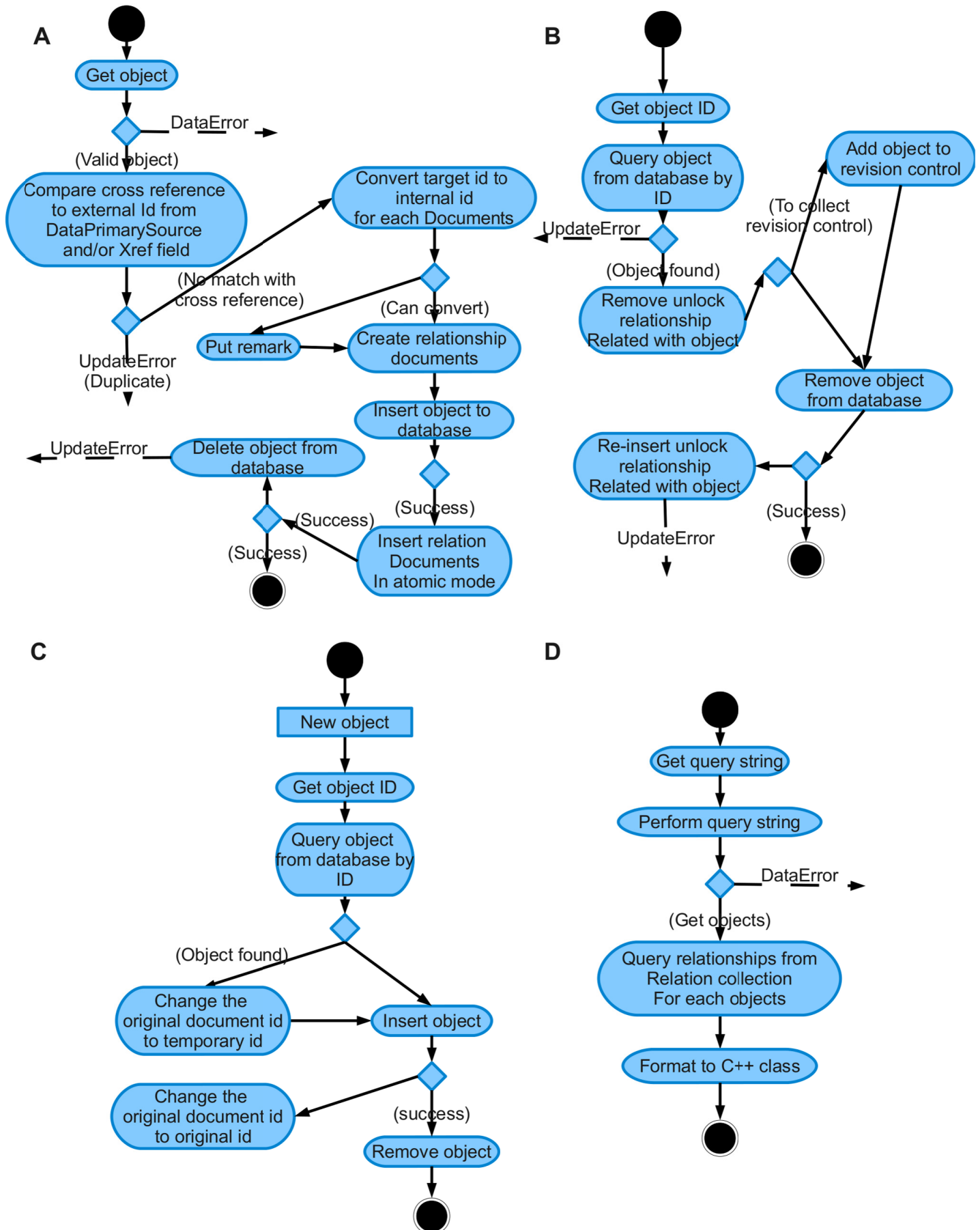

Supplement: Additional file 3 — Flow of activities in each function: A) Create; B) Delete; C) Update; and D) Read. [file 1751-0473-9-17-S3.pdf]
